# *FOXO1*, *PXK*, *PYCARD* and *SAMD9L* are differentially expressed by fibroblast-like cells in equine synovial membrane compared to joint capsule

**DOI:** 10.1186/s12917-017-1003-x

**Published:** 2017-04-14

**Authors:** Line Nymann Thomsen, Preben Dybdahl Thomsen, Alison Downing, Richard Talbot, Lise Charlotte Berg

**Affiliations:** 1 Department of Veterinary Clinical Sciences, University of Copenhagen, Hoejbakkegaards alle 5, 2630 Taastrup, Denmark; 2 Department of Veterinary and Animal Sciences, University of Copenhagen, Groennegaardsvej 7, 1870 Frederiksberg C, Denmark; 3 Edinburgh Genomics, Ashworth Laboratories, University of Edinburgh, Edinburgh, EH9 3FL UK

**Keywords:** Synovial membrane, Fibroblast-like synoviocytes, *FOXO1*, *PXK*, *PYCARD*, *SAMD9L*, Equine, Marker

## Abstract

**Background:**

The synovial membrane lines the luminal side of the joint capsule in synovial joints. It maintains joint homeostasis and plays a crucial role in equine joint pathology. When trauma or inflammation is induced in a joint, the synovial membrane influences progression of joint damage. Equine synovial membrane research is hampered by a lack of markers of fibroblast-like synoviocytes (FLS) to distinguish FLS from other fibroblast-like cells in musculoskeletal connective tissues. The aim of this study is to identify potential FLS markers of the equine synovial membrane using microarray to compare between gene expression in equine synovial membrane and the joint capsule in metacarpophalangeal joints.

**Results:**

Microarray analysis of tissues from 6 horses resulted in 1167 up-regulated genes in synovial membrane compared with joint capsule. Pathway analysis resulted in 241 candidate genes. Of these, 15 genes were selected for further confirmation as genes potentially expressed by fibroblast-like synoviocytes. Four genes: *FOXO1, PXK, PYCARD* and *SAMD9L* were confirmed in 9 horses by qPCR as differentially expressed in synovial membrane compared to joint capsule.

**Conclusions:**

In conclusion, *FOXO1*, *PXK*, *PYCARD* and *SAMD9L* were confirmed as differentially expressed in synovial membrane compared to joint capsule. These four genes are potential markers of fibroblast-like synoviocytes of the synovial membrane. As these genes are overexpressed in synovial membrane compared to joint capsule, these genes could shed light on synovial membrane physiology and its role in joint disease.

## Background

Horses are susceptible to joint disease and trauma leading to joint pathology. Joint pathology involves complex processes, and joint inflammation affects several tissues within the joint [1]. Regardless which intra-articular tissue type is first affected, the synovial membrane orchestrates and reinforces inflammatory responses of joints [2, 3]. Hence, the synovial membrane is key to enhance understanding of the pathophysiological processes within synovial joints.

In the healthy joint, the synovial membrane maintains joint homeostasis [4]. From its position as luminal lining of the joint capsule, the synovial membrane facilitates diffusion of plasma ultra-filtrate through synovial extracellular matrix and produces lubricating synovial additives [4]. Two cell types constitute the majority of the synovial membrane, macrophage-like synoviocytes (MLS) and fibroblast-like synoviocytes (FLS) [5]. MLS are macrophages resident to the joint [6, 7] which tend to distribute unevenly adjacent to the joint lumen [8] and to congregate at the top of synovial villi [6]. From this localisation within the synovial membrane, the MLS engulf foreign substances [6, 7]. MLS are characterized by expression of typical general resident macrophage-markers such as CD11b, CD14, CD68 and CD206 [9–11]. The fibroblast-like synoviocytes (FLS) are modified fibroblasts with cytoplasmatic processes reaching to the joint lumen. FLS produce and secrete lubricating additives to the synovial fluid and produce and maintain the synovial extracellular matrix (ECM). FLS are the dominating cell-type in the synovial intima (80%) and the fibroblastic lineage results in characteristic expression of general fibroblast markers such as vimentin and prolyl hydroxylase [12]. Due to FLS’s specific functions in the synovial lining [13], FLS differ from other fibroblasts, but there are only a few reports of selective markers of FLS differentiating them from other musculoskeletal fibroblasts. In humans, the hyaluronan precursor Uridine diphosphoglucose dehydrogenase (UDPGD) has been proposed as a specific marker of FLS [14–16]. In mice and humans, the cell contact mediating protein Cadherin-11, has been suggested as marker of FLS. Cadherin-11 is believed to mediate synovial lining integrity and to be implicated in synovial inflammation [3, 17]. None of these molecules have been investigated in horses and new equine FLS markers in the synovial membrane could elucidate the normal function of the synovial membrane and potentially contribute to unravel mechanisms behind the role of FLS in arthritic conditions. In addition, novel markers could improve the consistency and reproducibility of FLS based studies.

Thus, the aim of this study was to identify new markers of equine healthy FLS in the healthy joint. We compared the gene expression of the synovial membrane to the gene expression of the fibrous joint capsule using a microarray technique. This is a comparison of two tissues each containing different types of fibroblasts, the FLS of the synovial membrane and fibroblasts from the dense connective tissue of the joint capsule. This comparison was made to highlight differences in gene expression between the two types of cells and thus search out markers not previously explored. Based on the microarray data we used pathway analysis to select markers relevant to FLS, and we confirmed the selected target genes by qPCR to support general applicability of the markers.

## Methods

### Microarray experiment

#### Tissue samples

Synovial membrane and joint capsule tissue samples were collected from six horses of mixed breed (aged 15–25 years). The horses were euthanized at the Faculty of Health and Medical Sciences, University of Copenhagen according to regulations of Danish law for reasons unrelated to orthopaedic disorders in the metacarpophalangeal joints. The joint capsule was transected and the macroscopic appearance of the joint evaluated. Synovial membrane and joint capsule were excised from the entire fetlock joint to avoid site-specific variation within the joint. The synovial membrane was separated from the joint capsule by careful dissection. This method was verified by embedding dissected synovial membrane and joint capsule separately in paraffin and subsequently staining with Mayer’s Hematoxylin^1^ and Eosin^2^ prior to histologic evaluation. The separated exised tissue from the six horses generated a yield of 0.6–4.0 g of synovial membrane and joint capsule from each joint, which was snap frozen in liquid nitrogen. To evaluate for signs of acute inflammation a full thickness tissue sample was excised from the proximo-dorsal part of the joint, fixed in 4% paraformaldehyde^3^ in PBS,^4^ and embedded in paraffin. This sample was later sectioned and stained with Mayer’s Hematoxylin and Eosin and evaluated for histologic signs of acute inflammation. The histologic signs of acute inflammation evaluated were: Cellular infiltrates in general and in vascular surroundings and/or hyperplasia of the synovial membrane. Tissues from joints with macroscopic or cellular signs of inflammation were excluded from the study.

#### RNA extraction

For the microarray study, RNA was extracted using Trizol. Briefly, 1 ml Trizol^5^ was added to the tissue and the tissue was homogenised in a Fastprep^6^ homogeniser for 20 s at 4 m/s. Following 5 min incubation, 0.2 mL of 1-bromo-3-chloro propane^7^ was added for phase separation and the tubes were shaken vigorously by hand for 15 s. Then the samples were incubated for 2 min, and centrifuged at 12,000 *g* for 15 min. 500 μL of the aqueous upper phase was carefully aspirated and transferred to new RNAse free 1.5 mL tubes. The RNA was precipitated by adding 1 μL of linear polyacrylamide,^8^ gentle mixing and the addition of 500 μL isopropyl alcohol.^9^ Following 10 min incubation the tubes were centrifuged at 14,000 *g* for 10 min. The supernatant was carefully removed and the RNA pellet washed with 1 mL 75% Ethanol, the tubes inverted and centrifuged for 5 min at 14,000 *g*. After a second wash in 75% ethanol the RNA pellet was left to air dry until any residual liquid had evaporated, approximately 5 to 15 min. The RNA was re-dissolved in 20 μL of MilliQ water. The yield of RNA was quantified using a NanoDrop^10^ spectrophotometer (Labtech International Ltd., UK) and the quality assessed using an Agilent RNA 6000 nanochip on the Agilent Bioanalyser.^11^

#### Amplification of the RNA, aminoallyl incorporation and dye coupling

Total RNA was amplified using the MessageAmp™ II aRNA Amplification Kit,^12^ and spiked control RNA’s were added according to the Agilent Spike in Kit^13^ with 500 ng RNA and 5 μl Spike mix added to each well and the volume adjusted to 10 μl by adding nuclease-free water.

Reverse transcription of RNA was initiated by adding 10 μl of a reverse transcription mastermix containing reverse transcriptase, NTP and T7 Olio d(T) primer and incubated according to the manufacturer’s instructions. After 2 h at 42 °C the temperature was dropped to 16 ^o^ C and second strand synthesis started using a mixture of fresh NTP with DNAse and Ribonuclease H and incubated for a further 2 h. Double stranded cDNA was purified on a spin column before aRNA synthesis.

The aRNA was synthesized by in vitro transcription. In vitro transcription was performed by adding 16 μl cDNA to a mixture consisting of 3 μl 5-(3-aminoallyl)-UTP,^14^ 4 μl T7 ATP solution, 4 μl T7 CTP solution, 4 μl T7 GTP solution, 2 μl T7 UTP solution, 4 μl T7 10 Reaction Buffer and 4 μl Enzyme mix, according to manufacturer’s instructions. The aRNA was purified according to the MessageAmp™ II aRNA Amplification Kit protocol. The yield of the aRNA was assessed using a NanoDrop. The uniformity of the size of the aRNA was assessed using a RNA 6000 nanochip on the Agilent Bioanalyser.

Cy3^15^ dye was coupled to aminoallyl UTP (aaUTP) in the amplified aRNA, according to manufacturer’s instructions. The yield and specific activity of the eluate containing the purified labelling reactions was quantified using a Nanodrop TM Spectrophotometer (Labtech International Ltd., UK).

#### Fragmentation and hybridisation of the samples to the Agilent Array

Twelve Agilent equine microarrays were used in the study. Each microarray was hybridised with a single labelled RNA sample. The Cy 3 labelled aRNA (10 μl) was mixed with 10 μl blocking Agent, 31.8 μl nuclease free water, and 2.2 μl fragmentation buffer,^16^ and incubated at 60 °C for 30 min. The reaction was stopped by adding GE hybridization buffer. The samples were loaded onto the array and the sides were placed in rotisserie in a hybridization oven at 65 °C for 17 h.

#### Scanning of array and subsequent data analysis

After hybridization, the arrays were scanned on a 4200A axon scanner^17^ using autoPMT and the appropriate GAL file.

Image files were imported using Agilent Feature extraction software^18^ and Grids were manually fitted to the arrays according to the manual. The extracted intensities were imported into Partek Genomics Suite^19^ where they were analysed using the gene expression workflow. The array data was normalised using a quantile normalisation where the software calculates the distribution model from the data files and normalize the probes according to the calculated distribution model. Analysis of variance was performed on the normalized data, with Bonferroni’s correction applied.

The Agilent equine microarray was not fully annotated, but the annotation was improved by importing ENS numbers and LOC numbers from AgBase [18, 19]. A list of the accession numbers for each non-annotated probe was prepared and used to query the AgBase database using the online search tool.

The significantly up-regulated genes were imported into IPA [20]. Significantly overexpressed canonical pathways were explored.

### Confimation of microarray data

#### Tissue samples

The microarray results were confirmed using qPCR on synovial membrane and joint capsule in nine horses (three of the horses from the microarray experiment and six additional horses of mixed breed (aged 2–22 years)). Equine synovial membrane and joint capsule was excised and processed as described previously, and presence of joint pathology was evaluated as previously described.

#### RNA extraction

RNA from the tissue samples was extracted using the Promega SV total RNA Isolation system,^20^ according to manufacturer’s instructions.

The yield of RNA was measured using a NanoDrop TM Spectrophotometer^21^ and the quality assessed by using an Agilent Bioanalyser.^22^

Subsequently, the RNA was transcribed to cDNA by adding 5× MMLV RT buffer,^23^ dNTP^24^ (10 nM), random hexamer primer^25^ (2 μg/μL), oligo(dt)^26^ (0.5 μg/μL), RNasin RNase inhibitor^27^ (40 U/μL) and MMLV reverse transcriptase enzyme^28^ (200 U/ μL) to 800 ng RNA diluted in H_2_O to a 25 μL reaction volume. The cDNA was transcribed at 25 °C for 10 min, 42 °C for 60 min, 95^o^ C for 5 min. The cDNA was stored at −20 ^o^ C and subsequently diluted to a concentration of 4 ng/μL for all samples.

#### Selection of reference genes

The following reference genes were tested for qPCR: 18S ribosomal RNA (18 S), glyceraldehyde-3-phosphate dehydrogenase (GAPDH), β-actin, mitochondrial ribosomal protein S7 (MRPS7), and 5-aminoimidazole-4-carboxamide ribonucleotide formyltransferase /IMP cyclohydrolase (ATIC). 18S was chosen as reference gene.

#### Primer design, candidate genes

The equine gene sequences corresponding to the 15 selected candidate genes were found in Ensembl [21]. If multiple transcript variants existed, primers were designed in conserved sequences. The transcript sequences (mRNA sequences) were inserted in the programme Primer 3 [22]. The chosen primer sequences were subsequently screened against the horse genome using BLAST ref. [23] to ensure primer specificity. Amplified gene products were confirmed for specificity through sequencing. Only primer sets with amplification efficiency between 1.8–2.2 were used. Primer sequences are listed in Table 1.

**Table 1:** Primers used to confirm the expression of candidate genes found in microarray experiment

Target	Accession	Forward primer	Reverse primer	Product size (base-pair)
*ATXN1*	[Genbank: XM_001496252]	CCCAAAAGCGAGAACTTCAG	TCCGTTTTCAAGTCCTCCAC	206
*COL11A1*	[Genbank: XM_001918115]	GGCAATTCCATTAACATGGTG	TTGATCCCAGGAATCGAAGT	151
*COL28A1*	[Genbank: XM_001494969]	AGACTCCGCTAGAGCTGCTG	AAGTCGTCCTTGCTGGAGAA	202
*FSTL1*	[Genbank: XM_001500510]	ACAAGAGGCCTGTGTGTGG	TGTCCGTCATAATCGACCTG	130
*FOXO1*	[Genbank: XM_005601179]	CCAGCCCAAACTACCAAAAA	AACACATTCTGGCCAAGGAC	241
*GALNT3*	[Genbank: XM_001496049]	CCTTGCTCTGTTGTTGGACA	TGCTGCATCTGTGTTTCTCC	153
*GRN*	[Genbank: XM_001489741]	TGTGAGGAGGGACTGAGGAC	TTGTTACGTGGCTTTCACCA	207
*ITPR3*	[Genbank: XM_005604053]	GCAGTTTGGGATGATGCAGT	CTGGGCTCTCGGTTCTTG	159
*NF1*	[Genbank: XM_005597955]	TGGCCCTGTACATGTTTCTG	TTGCTGACAGACGCAAATTC	172
*PXK*	[Genbank: XM_008519676]	CATTACCTCCACCTCCTCCA	GATCACAGGTTTCGGCTTTC	180
*PYCARD*	[Genbank: XM_001500509]	CCATCCTAGAGGCACTGGAA	CTCCGTACGCCTCCAGATAG	177
*RGL2*	[Genbank: XM_001497200]	GGATGGAGCTTCACACGATT	CAGGATGGCTACCCACATCT	235
*SAMD9L*	[Genbank: XM_005609186]	GAACCGGAAAACGTCTGTGT	GGGAGAAAGTCGGTGCATTA	171
*SOS1*	[Genbank: XM_005600032]	CACGAGAACCTGTGAGGACA	AAGTGCTTTGTCGAGGAGGA	249
*TIMP3*	[Genbank:NM_001081870]	CCTGCTACTACCTGCCTTGC	GGTCTGTGGCATTGATGATG	186

#### qPCR

The quantitative real time polymerase chain reaction (qPCR) was performed in 96 wells plates on Lightcycler 480 using SYBR Green I^29^ detection. Each reaction consisted of 10 μl containing 2 μl cDNA, 1 μl forward primer, 1 μl reverse primer, 5 μl master mix and 1 μl H_2_O. All samples were tested for genomic contamination by the use of an intron-spanning primer set unrelated to the study.

Gene expression of the target genes was calibrated between qPCR runs, and all samples were run in triplicate. The relative gene expression was calculated using Pfaffl’s methods [24, 25] and normalised to the reference gene 18S. The relative normalised gene expression was analysed between synovial membrane and joint capsule. Mean normalised gene expression was analysed using Student’s t test in SAS JMP software.^30^ The assumption of normal distribution was not met, but the assumption of equal variances was met for 10 of 15 groups (Table 2).

**Table 2:** Real-time polymerase chain reaction results for the 15 selected candidate genes

Target gene	Synovial membrane	Joint capsule
*ATXN1*	0.40 ± 0.30^b^	0.43 ± 0.30
*COL11A1*	1.35 ± 1.81	0.43 ± 0.47
*COL28A1*	0.36 ± 0.47^b^	0.17 ± 0.28
*FSTL1*	7.75 ± 9.99^a^	0.27 ± 0.32
*FOXO1*	3.35 ± 2.97*	0.93 ± 1.15
*GALNT3*	0.99 ± 1.70	0.15 ± 0.17
*GRN*	7.05 ± 7.63^a^	1.44 ± 1.66
*ITPR3*	1.90 ± 1.11^b^	1.07 ± 0.83
*NF1*	2.96 ± 3.42	5.60 ± 5.96
*PXK*	7.49 ± 7.69*	0.49 ± 0.62
*PYCARD*	2.70 ± 2.43*	0.34 ± 0,29
*RGL2A*	2.06 ± 1.70^b^	1.37 ± 1,66
*SAMD9L*	5.39 ± 4.79*	0.45 ± 0.45
*SOS1*	1.83 ± 2.19^b^	0.77 ± 0.77
*TIMP3*	32.26 ± 46.34^a^	2.95 ± 4.06

Results are reported as expression of target gene relative to expression of 18S, the expression is furthermore normalised to a calibrator. The reported results are mean expression ± standard deviation. Difference between groups was tested with a Student’s t-test. *Significant difference between Synovial membrane and Joint capsule, *p* ≤ 0.05. ^a^Differential expression in synovial membrane compared to joint capsule *p* value ≤0.10. ^b^Test of equal variances in the groups was not significant

## Results

### Microarray analysis

The microarray analysis of variance resulted in 2995 probes from 1907 genes significantly differentially expressed in synovial membrane compared to joint capsule (*p* ≤ 0.05). Selection of the genes up-regulated in synovial membrane rendered 1167 genes.

Ingenuity Pathway Analysis (IPA) [20] was used to select relevant up-regulated genes in synovial membrane as potential FLS candidate genes. The top biological functions explored were: ‘Connective tissue disorders’, ‘Inflammatory disease’ and ‘Skeletal and Muscular disorders’. Within ‘Connective tissue disorder’, the functions annotations of *rheumatic disease*, *arthritis*, and *rheumatoid arthritis* were analysed. In addition, we explored the molecular and cellular function categories: ‘Tissue morphology’, ‘Small molecule biochemistry’, ‘Cell morphology’, and ‘Cellular movement’.

A total of 241 genes were evaluated as potential candidate genes. Genes were included in the final candidate gene-list if they related to FLS function or cellular origin (mesenchymal cells). This led to a sub-classification of the genes into the following groups: genes potentially relating to FLS function, genes potentially relating to FLS cell morphology, genes relating to mesenchymal cells, or adverse genes (genes relevant to diseases involving mesenchymal cells).

This led to selection of 15 candidate genes. The fold change and the *p*-values of the 15 candidate genes in the microarray study are presented in Table 3.

**Table 3:** Microarray Fold change and p-values for the 15 selected candidate genes

Gene symbol	Function	Fold change	*p*-value ANOVA
*ATXN1*	Embryonal ECM remodelling	2.48	2.96E-03
*COL11A1*	Collagen in ECM	11.82	2.93E-04
*COL28A1*	Collagen in vascular ECM	21.29	1.63E-04
*FSTL1*	Unknown, embryonal mesenchymal location	4.10	2.59E-03
*FOXO1*	Transcription factor, function in osteoblast homeostasis	2.33	2,50E-05
*GALNT3*	Glycosylation, golgi complex	5.65	8.88E-07
*GRN*	Regulate cell growth	1.99	5.49E-06
*ITPR3*	Second messenger, possible role in exocrine secretion	3.09	6.64E-04
*NF1*	Regulate cell growth	3.27	4.35E-04
*PXK*	A functionel sorting nexin	4.81	1.72E-04
*PYCARD*	Function in inflammasomes	3.70	8.63E-05
*RGL2A*	Protein transport in cells	1.58	1.64E-05
*SAMD9L*	Protein-protein interaction	4.88	5.80E-04
*SOS1*	Promotes reorganization of actin cytoskeleton	2.90	1.20E-03
*TIMP3*	Metalloproteinase inhibitor	1.75	9.79E-04

*ATXN1* ataxin 1, *COL11A1* collagen XI α1, *COL28A1* collagen XXVIII α1, *FOXO1* forkhead box O1, *FSTL1* follistatin-like 1, *GALNT3* UDP-N-acetyl-alpha-D-galactosamine:polypeptide N-acetylgalactosaminyltransferase 3 (GalNac-T3), *GRN* granulin, *ITPR3* inositol 1,4,5-trisphosphate receptor, type 3, *NF1* neurofibromin 1, *PXK* PX domain containing serine/threonine kinase, *PYCARD* PYD and CARD domain containing, *RGL2* RGL2, ral guanine nucleotide dissociation stimulator-like 2, *SAMD9L* sterile alpha motif domain containing 9-like, *SOS1* son of sevenless homolog 1 (Drosophila), *TIMP3* TIMP metallopeptidase inhibitor 3

### Real-time PCR confirmation of candidate genes

The relative quantification analysis detected significantly different gene expression of *FOXO1*, *PXK*, *PYCARD* and *SAMD9L* at significance level *p* ≤ 0.05 between synovial membrane and joint capsule (Table 2). In addition, the genes FSTL1, GRN and TIMP3 were expressed differently in synovial membrane compared to joint capsule at significance level *p* ≤ 0.10 (Table 2).

## Discussion

In this microarray study, we investigated new markers of the equine synovial membrane by comparing gene expression of synovial membrane to the gene expression of the joint capsule. Of 1167 genes up-regulated in the synovial membrane, we evaluated 241 genes with potential relevance to FLS to search for FLS markers. Genes were included in the final candidate gene-list, if the genes could be related to mesenchymal origin or FLS function. By careful selection of relevant candidate genes, the final candidate gene-list was reduced to 15 genes.

We reduced variations in probe set expression and gene expression between horses by normalisation between microarrays. Barrey et al., 2009 used data mining software to identify networks and associated genes in an equine microarray study [26]. In the present study relevant networks were visualised using Ingenuity Pathway Analysis (IPA), and the filtering process applied related to IPA results and a subjective selection of putative candidate genes. The subjective filtering process involved exclusion of genes with known expression in macrophages, endothelial cells or neurons, and genes with known ubiquitous gene expression. Genes with known mesenchymal expression were included in the candidate gene-list, even though this could include genes simultaneously expressed in adipocytes, connective tissue fibroblasts, chondrocytes and osteoblasts. This filtering process is likely to have excluded some potential novel markers of FLS, and it is highly relevant to further explore the gene list of up-regulated genes in synovial membrane.

Inter-horse variation and anatomical location may affect the results. Huang et al.*,* 2008 compared ten different types of tissue to cartilage to identify novel markers of cartilage. Most of the samples in the study by Huang et al.*,* 2008 were taken from the same horse [27]. Rinn et al.*,* 2006 showed different gene expression in fibroblasts from various anatomical locations [28]. We aimed to minimize anatomical variation by excising samples from only one joint in a total of 12 horses. However, our candidate gene list may include genes differentially expressed merely as a result of the anatomical position of the cells instead of being specific to synovial membrane fibroblast-like cells.

It has also been shown that age, gender, breed, and activity level can contribute to variation in levels of biomarkers in the joint or serum in horses with osteoarthritis [29–31]. In our study, we included mixed breed, gender, and age searching for candidate genes of general applicability. It was not the aim of this study to investigate the influence of breed, gender or age on the gene expression of the synovial membrane compared to the joint capsule which would call for a larger sample size.

The differential gene expression of *FOXO1*, *PXK*, *PYCARD* and *SAMD9L* was confirmed using qPCR. In addition, the genes: *FSTL1, GRN* and *TIMP3* showed a tendency towards higher gene expression in synovial membrane compared to joint capsule.

The genes presented here represent possible new markers of the synovial membrane. Knowledge of function of the four significantly differentially expressed genes in horses is limited. But in humans, the forkhead transcription factor *FOXO1* has turned out to be a regulator of glucose expenditure in cells and plays a role in regulation of glucose homeostasis in osteoblasts [32]. This suggests that *FOXO1* may regulate glucose homeostasis in FLS. Human *FOXO1* is also expressed in activated macrophages in lung tissue [33]. It could therefore be expressed in activated macrophages in joints, but a study in human rheumatoid arthritis synovial membrane showed that *FOXO1* expression occur mainly in FLS and only occasionally in MLS, supporting the use of *FOXO1* as a marker of FLS [34].

*PXK* encodes a phox homology domain, which is suggested as a sorting nexin localised in endosomes in cells and possibly interacting with actin. These functions suggest that *PXK* is involved in sorting processes in the cells and possibly in receptor trafficking [35].

*PYCARD* encodes for a protein in inflammasomes, a multiprotein complex which contributes to initiating the inflammatory process by activation of pro-caspase 1, which further leads to production of inflammatory cytokines [36]. Evidence indicates that the protein encoded by *PYCARD* is expressed in both FLS and MLS [37], but not at a high level in joint capsule fibroblasts according to the present study. Further studies are warranted to clarify if the expression of *PYCARD* is found in both FLS and MLS of the synovial membrane.

Expression of the gene *SAMD9L* has been located to a variety of tissues, but the exact function of the gene transcript is unknown. A recent study has investigated proliferation depressing effects of *SAMD9L*, and *SAMD9L* expression was reduced in tumours [38]. Thus, if *SAMD9L* is specifically expressed by FLS in synovial membrane and the gene has a proliferation depressing effect, the expression of the gene in FLS from joints with inflammatory arthritis or rheumatoid arthritis should be investigated. It is worth noting that neither of the human FLS markers suggested in the literature, UDPGD nor cadherin 11, appeared on the list of significantly up-regulated genes in our microarray study.

## Conclusions

In conclusion, the genes *FOXO1*, *PXK*, *PYCARD* and *SAMD9L* were confirmed as differentially expressed between synovial membrane and joint capsule. We suggest inclusion of the genes *FOXO1, PXK, PYCARD* and *SAMD9L* as potential markers of FLS in future studies of the equine synovial membrane.

## Abbreviations

ECM: Extracellular matrix
FLS: Fibroblast-like synoviocytes
IPA: Ingenuity pathway analysis
MLS: Macrophage-like synoviocytes
PBS: Phosphate- buffered saline solution
qPCR: Quantitative polymerase chain reaction
UDPGD: Uridine diphosphoglucose dehydrogenase

## Footnotes

1 VWR & Bie & Berntsen A/S, Herlev, Denmark

2 Merck, Darmstadt, Germany

3 VWR & Bie & Berntsen A/S, Herlev, Denmark

4 University of Copenhagen, Copenhagen, Denmark

5 Life Technologies Ltd., Paisley UK.

6 Fisher Scientific UK Ltd.,Loughborough UK

7 Sigma Aldrich Ltd. Dorset UK

8 Life Technologies Ltd., Paisley UK

9 Fisher Scientific UK Ltd.,Loughborough UK

10 Labtech International Ltd., Uckfield, UK

11 Agilent Technologies UK Limited, Stockport UK

12 Life Technologies Ltd., Paisley UK

13 Agilent Technologies UK Limited, Stockport UK

14 Life Technologies Ltd., Paisley UK

15 GE Healthcare Life Sciences, Little Chalfont, UK

16 Agilent Technologies UK Limited, Stockport UK

17 Molecular Devices (UK), Wokingham, UK

18 Molecular Devices (UK), Wokingham, UK

19 Partek Incorporated. Saint Louis, USA

20 Promega Biotech AS, Nacka, Sweden

21 Thermo Scientific, Wilmington, US

22 Agilent Technologies Denmark Aps, Hørsholm, Denmark

23 Promega Biotech, Nacka, Sweden

24 Promega Biotech, Nacka, Sweden

25 Promega Biotech, Nacka, Sweden

26 Promega Biotech, Nacka, Sweden

27 Promega Biotech, Nacka, Sweden

28 Promega Biotech, Nacka, Sweden

29 Roche, Hvidovre, Denmark

30 SAS Institute Inc., North Carolina, USA
